# Cationic Liposomes Carrying siRNA: Impact of Lipid Composition on Physicochemical Properties, Cytotoxicity and Endosomal Escape

**DOI:** 10.3390/nano8050270

**Published:** 2018-04-24

**Authors:** Anna Lechanteur, Vincent Sanna, Amandine Duchemin, Brigitte Evrard, Denis Mottet, Géraldine Piel

**Affiliations:** 1 Laboratory of Pharmaceutical Technology and Biopharmacy, CIRM, University of Liège, CHU Bat B36 Tour 4, +2, 1 avenue de l’Hopital, 4000 Liège, Belgium; vincent.sanna@student.ulg.ac.be (V.S.); b.evrard@uliege.be (B.E.); geraldine.piel@uliege.be (G.P.); 2 Laboratory of Protein Signaling and Interactions, GIGA-Molecular Biology of Diseases, University of Liège, 4000 Liège, Belgium; amandine.duchemin@student.uliege.be (A.D.); dmottet@uliege.be (D.M.)

**Keywords:** liposome, siRNA, Cellular uptake: lipids, cytoxiciy

## Abstract

In recent year, cationic liposomes have gained a lot of attention for siRNA delivery. Despite this, intracellular barriers as endosomal escape and cytosolic delivery of siRNA still represent a challeng, as well as the cytotoxicity due to cationic lipids. To address these issues, we developed four liposomal formulations, composed of two different cationic lipids (DOTAP and DC-Cholesterol) and different ratio of co-lipids (cholesterol and DOPE). The objective is to dissect these impacts on siRNA efficacy and cytotoxicity. Liposomes were complexed to siRNA at six different N/P molar ratios, physico-chemical properties were characterized, and consequently, N/P 2.5, 5 and 10 were selected for in vitro experiments. We have shown that cytotoxicity is influenced by the N/P ratio, the concentration of cationic lipid, as well as the nature of the cationic lipid. For instance, cell viability decreased by 70% with liposomes composed of DOTAP/Cholesterol/DOPE 1/0.75/0.5 at a N/P ratio 10, whereas the same formulation at a N/P ratio of 2.5 was safe. Interestingly, we have observed differences in terms of mRNA knock-down efficiency, whereas the transfection rate was quite similar for each formulation. Liposomes containing 50% of DOPE induced a mRNA silencing of around 80%. This study allowed us to highlight crucial parameters in order to develop lipoplexes which are safe, and which induce an efficient intracytoplasmic release of siRNA.

## 1. Introduction

Cationic liposomes have been classified as one of the most attractive nanocarriers for small interference RNA (siRNA) delivery [1,2,3]. The RNA interference (RNAi) mechanism induced by siRNA holds high interest in treating human diseases—especially in cancers—since siRNA can selectively target and repress a specific gene associated with pathologies [4]. Among twelve ongoing clinical trials involving gene delivery, ten nanocarriers are made with lipids which demonstrate the high potential of lipidic nanovectors for siRNA delivery [5]. Lipidic nanoparticles offer multiple structure conformation possibilities once associated to siRNA like lipoplexes, stable nucleic-acid-lipid particles (SNALPs), lipopolyplexes, and membrane/core nanoparticles (MCNPs) [6].

Among the biological barriers, lipoplexes have to cross various intracellular barriers to be efficient as the cell adhesion, cellular uptake, the endosomal escape before lysosomal degradation, and finally the cytosolic dissociation of the complex [7,8]. Furthermore, their toxicity is still a hurdle to their utilization in therapy [9]. Liposomes engineered for siRNA delivery are basically made of two types of lipids: cationic lipids (CL^+^) and neutral pH-sensible or helper lipids. CL^+^ are essential for electrostatic interactions with anionic siRNA and for cell membrane interaction, and are classified as monovalent CL^+^ (DOTMA, DOTAP, DIMRIE, …), multivalent CL^+^ (DOGS, DOSPA) or cholesterol-derivatives CL^+^ (DC-Cholesterol) [2,3,10]. Neutral lipids like DOPE and DOPC are necessary for the destabilization of the lipoplexes in endosomes due to a conformation shift at acidic pH [7,11]. Depending on phospholipid structural aspect, such as head group, aliphatic chain or linker between them, the lipid proportion and the molar ratio between lipids and siRNA (N/P ratio) confer different characteristics to the lipids/siRNA complex.

The main goal of this study is to better understand which lipids influence both effectiveness and toxicity of lipoplexes formulations in vitro. Based on previous results [12,13,14], we decided to vary four parameters as the nature and the quantity of the CL^+^, the quantity of helper lipids, and the N/P molar ratio. The formulation DOTAP/Chol/DOPE 1/0.75/0.5 has previously been investigated by our group for vaginal administration of siRNA. We demonstrated that this liposome associated to polyethylene glycol (PEG) and complexed to siRNA at a N/P molar ratio of 2.5 is a good candidate for cervical cancer treatment by vaginal application [12,14]. However, this cationic formulation induced high toxicity on SiHa and CaSki cells, two HPV16 positive cell lines. We highlighted that the PEGylation can decrease the surface charge, and therefore, decrease the toxicity [13].

In the present study, we evaluated the impact of various factors on the siRNA in vitro efficacy and on the potential toxic effect. The transfection efficiency and the endosomal escape as a function of formulations were dissected. Compared to the first formulation DOTAP/Chol/DOPE 1/0.75/0.5, the ratio between lipids in the second formulation was adapted in order to study the impact of a higher proportion of DOTAP and of a lower of cholesterol (DOTAP/Chol/DOPE 1/0.5/0.5; Formulation 2). To observe the influence of the percentage of the neutral lipid DOPE, the formulation DOTAP/DOPE 1/1 has been also developed (Formulation 3). Finally, the nature of the CL^+^ was modified and DC-Chol was used instead of DOTAP (DC-Chol/DOPE 1/1; Formulation 4). Zhang et al. described that this lipid ratio between DC-Chol and DOPE is optimal for siRNA delivery [15]. The understanding of lipids influence is expected to facilitate the design of liposomes for efficient and safe siRNA delivery.

## 2. Results

### 2.1. Characterization of Lipoplexes

The size, the polydispersity index (PDI) and the surface charge were measured for the four formulations (DOTAP/Chol/DOPE 1/0.75/0.5; DOTAP/Chol/DOPE 1/0.5/0.5; DOTAP/ DOPE 1/1; DC-Chol/DOPE 1/1) without or with siRNA at six different N/P molar ratios (from 0.5 to 10). Physico-chemical properties of unloaded liposomal formulations are shown in Table 1.

All formulations present a Z-average diameter around 170 nm with PDI lower than 0.2. Moreover, the surface charge are close to +55 mV, which is logical since the DOTAP and DC-Chol have a net positive charge at physiological pH: DOTAP is composed of a quaternary ammonium permanently charged and the DC-Chol has a titrable tertiary amine group of pKa of 7.8 [16].

**Figure 1:**
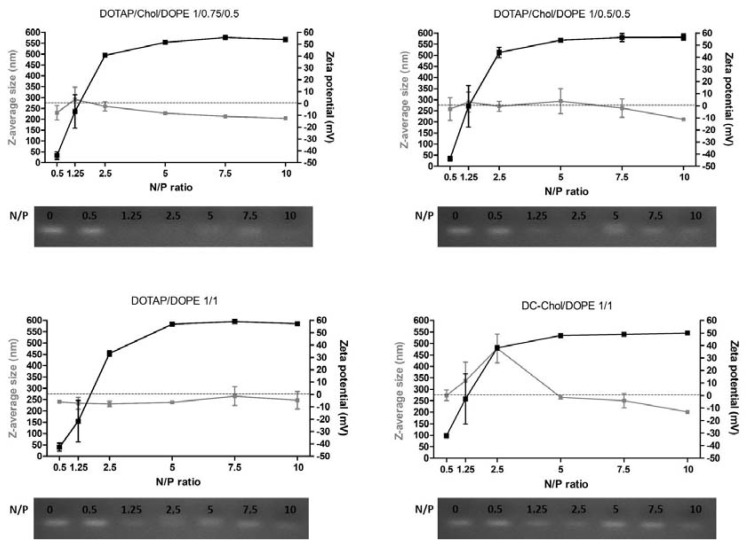
Z-average diameter (nm) and Zeta potential (mV) values of lipoplexes at different N/P molar ratios. Formulations DOTAP/Chol/DOPE 1/0.75/0.5, DOTAP/Chol/DOPE 1/0.5/0.5, DOTAP/DOPE 1/1 and DC-Chol/DOPE 1/1 were complexed to siCR at 100nM at N/P ratios of 0.5, 1.25, 2.5, 5, 7.5 and 10 (*n* = 4). The siRNA binding ability of lipoplexes was also evaluated by agarose gel retardation assay for all same formulations. The N/P 0 represents free siRNA.

Each liposomal formulation has been complexed to siRNA at N/P molar ratios of 0.5, 1.25, 2.5, 5, 7.5 and 10. Concerning the size, Figure 1 shows that, concerning formulations 1, 2 and 3, the Z-average diameter range is between 200 and 250 nm, irrespective of the N/P ratio. For these formulations, PDI values are between 0.17 and 0.27 (Figure S1). Formulation 4 made with the cationic DC-Chol lipid shows bigger size (201–478 nm), as well as a higher PDI value.

In terms of surface charge, all formulations follow the same profile in function of the N/P ratio. At low N/P ratio (0.5 and 1.25), particles are negatively charged whereas, the charge proportionally increased with the increase of N/P ratio. Some differences have been observed depending on formulations at N/P ratios of 2.5, 5 and 10. Indeed, at N/P 2.5, formulations with higher proportion of DOPE, and thus less CL^+^ (formulation 3 and 4), have a lower positive surface charge. Moreover, at N/P 5 and N/P 10, the DC-Chol provides a lower surface charge compared to the DOTAP lipid.

The complexation efficiency between cationic liposomes and anionic siRNA has been evaluated by gel electrophoresis retardation assay. The N/P 0 represents free siRNA. For all formulations, the N/P 0.5 does not allow the complexation of siRNA, since spots corresponding to free siRNA were observed. Complexation starts at N/P ratio 1.25, and complete complexation is observed from N/P 10. 

Based on present and previous results [12], N/P molar ratios of 2.5, 5 and 10 were selected for further cell comparative experiments. 

### 2.2. Cytotoxicity of Lipoplexes

**Figure 2:**
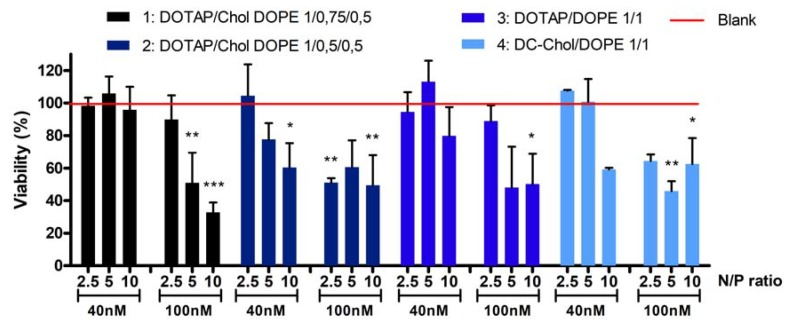
Cell viability of A549 cell lines treated during 24 h with liposomes complexed to inactive siRNA (siCR) at different N/P molar ratios at siRNA concentrations of 40 and 100 nM (*n* = 3). One-way ANOVA, post test Dunnett’s, *p* < 0.1 (*), *p* < 0.01 (**) and *p* < 0.001 (***) compared to untreated cells (Blank).

Two different concentrations of siRNA were tested: 40 and 100 nM. Therefore, the quantity of liposomes corresponding to a specific N/P ratio was also adapted meaning that the higher the concentration of siRNA and N/P ratio, the greater the increase in the amount of liposomes. A549 cells were treated during 24 h and the viability was evaluated in comparison to untreated cells. Figure 2 shows the cell viability after treatment with the four formulations.

At a 40nM siRNA concentration, no cytotoxicity is observed (cell viability is between 95% and 113%) for all formulations at N/P 2.5 and 5 except for the formulation 2 at N/P 5 (88% of cell viability). At N/P ratio 10, cytotoxicity depends on formulation type: cell viability is around 96%, 60%, 80% and 59%, respectively for formulations 1, 2, 3 and 4. At a 100 nM siRNA concentration and N/P ratio 2.5, formulations 1 and 3 are safe, since the viability is respectively 89% and 90%. However, all other formulations at this concentration induced a significant decrease of cell viability, ranging from 33 to 65%. Considering these toxicity results, a concentration of 40 nM siRNA was selected for further experiments.

### 2.3. Cellular Uptake of Lipoplexes

**Figure 3:**
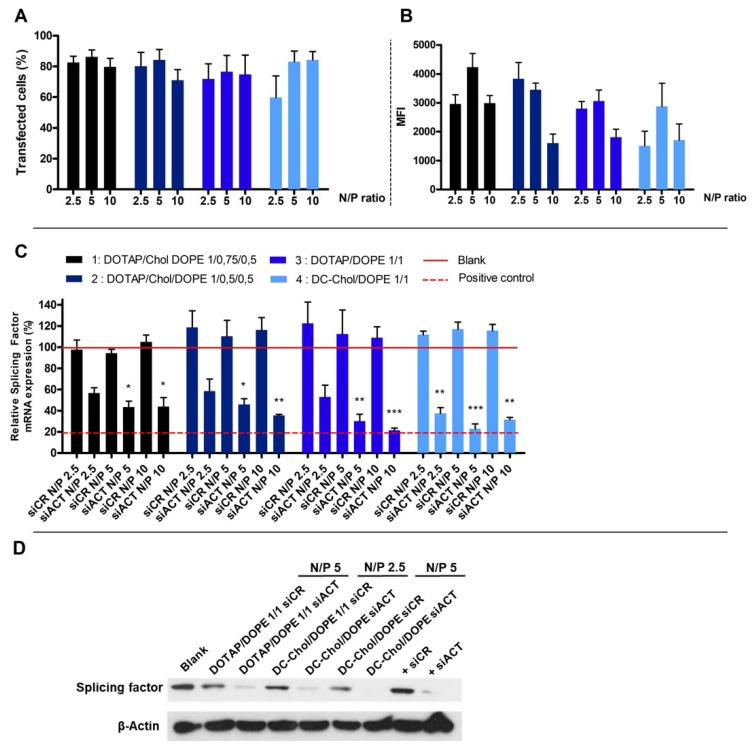
(**A**) Percentage of transfected A549 cells after the treatment by lipoplexes at different N/P ratios at siRNA concentration of 40 nM (4 h). Cells were post-treated with Trypan Blue solution; (**B**) Mean Fluorescence Intensity (MFI) was determined by flow cytometry 4 h after the treatment (*n* = 3). (**C**) Silencing efficiency of a splicing factor mRNA with lipoplexes carrying the corresponding siRNA targeting this splicing factor (siACT) or a siRNA control (siCR). A549 cells were transfected during 48 h by lipoplexes with 40nM of siRNA (*n* = 4). The calcium phosphate was used as a transfection positive control following the same procedure. One-way ANOVA, post test Dunnett’s, *p* < 0.1 (*), *p* < 0.01 (**) and *p* < 0.001 (***) compared to untreated cells (Blank); (**D**) Silencing efficiency of the targeted splicing factor protein with selected lipoplexes carrying the corresponding siRNA directed against this splicing factor (siACT) or a siRNA control (siCR). A549 cells were transfected during 72 h by lipoplexes with 40 nM of siRNA. The calcium phosphate was used as a transfection positive control (+). β-Actin was used as loading control.

The cellular uptake of lipoplexes was evaluated by flow cytometry following the quenching of external fluorescence. Two different parameters were evaluated with the flow cytometer as the percentage of transfected cells and the Mean Fluorescence Intensity (MFI). We showed that the post-treatment of cells with Trypan Blue^®^ strongly decrease the MFI (Figure S2). Furthermore, the more the N/P ratio of lipoplexes increases, the more the quantity of siCR-FITC blocked outside the cell increases. Indeed, ±35% and ±75% of siRNA do not enter into the cells when lipoplexes at N/P ratios 2.5 or 10 were used. Figure 3A,B show the percentage of transfected cells and the MFI after treatment by Trypan Blue^®^. The percentage of transfected cells is higher than 70%, whatever the type of liposomes and the N/P ratios, except for the formulation 4 (DC-Chol/DOPE 1/1) at N/P ratio of 2.5. The MFI values are very high, ranging from 1511 to 4233. 

### 2.4. Cytosolic siRNA and Gene Silencing

The gene silencing was evaluated for each type of lipoplexes by qRT-PCR using a siRNA directed against a splicing sactor (siACT) (Figure 3C). We observed that, compared to a control siRNA (siCR), the siACT induces an intense mRNA knock-down, since it remained at a maximum of 58% of the mRNA. We have seen that the N/P ratio of 2.5 does not allow a high extinction rate except for the formulation 4 to occur. Six types of lipoplexes induced an mRNA extinction comparable to the one induced by the calcium phosphate, the transfection positive control. Indeed, formulation 2 (DOTAP/Chol/DOPE 1/0.5/0.5) at N/P 10, formulation 3 (DOTAP/DOPE 1/1) at N/P 5 or 10 and formulation 4 (DC-Chol/DOPE 1/1) at N/P 2.5, 5 or 10 repressed the mRNA of 65%, 65%, 70%, 79%, 63%, 77% and 69%, respectively.

According to cytotoxicity and mRNA knock down results, the formulation 3 (DOTAP/DOPE 1/1) at N/P ratio of 5 and formulation 4 (DC-Chol/DOPE 1/1) at N/P ratios of 2.5 and 5 were selected. The gene silencing of these selected formulations was confirmed by Western Blot. Figure 3D shows an intense protein knockdown when siACT was used compared to siCR.

## 3. Discussion

Cationic liposomes developed for siRNA delivery are mainly composed of CL^+^ and co-lipids. Four cationic liposomes made with DOTAP or DC-Chol as CL^+^ and, DOPE and/or Chol as co-lipids were developed to explore their role in terms of siRNA effectiveness and cytotoxicity. Formulations were complexed to siRNA at six different N/P ratios (Table 2), and the influence of the ratio between CL^+^ and anionic siRNA was evaluated as well.

We observed that the physico-chemical properties of the four formulations were not different, whatever the lipid composition and the N/P ratio. The sizes are close to 200 nm, which yielded a good interaction between nucleic acids and cationic vesicles. This size is also described as appropriate for gene delivery [17]. In terms of surface charge, typical sigmoidal plots of ζ-potential are obtained when liposomes are complexed to siRNA at increasing N/P ratios, but very slight differences are observed in function of lipid composition. From N/P ratio 5, a plateau is reached around 50 mV. Concerning the CL^+^ DOTAP, we observed equal ζ-potential whatever its percentage within formulations, which can be explained by a saturation effect close to +50 mV. This is in accordance with recent findings of Kolasinac et al. who showed that from 50% of DOTAP in formulations, the surface charge was saturated at +40 mV [18].

Multiple parameters influence the toxicity, such as the nature of the CL^+^, the surface charge, the cell type or the cell density [19]. Concerning the nature of the CL^+^, Lv et al. explained that the positive charged headgroup, the hydrophobic chains, as well as the linker, have effects on toxicity levels [9]. Monovalent lipids with a biodegradable ester linker as DOTAP are supposed to be less toxic than polycationic lipid or ether linker [2]. Moreover, lipids derived from cholesterol like DC-Chol are known to be compatible with the biological membrane. In this study, we have highlighted different parameters that impact the cell viability. The first is the concentration of siRNA and the N/P ratio (Figure 2). The higher the concentration and/or the N/P ratio, the more the cell viability decreases irrespectively of formulation. Masotti et al. have also shown a gradual decrease of cell viability when N/P ratio increases [20]. The second highlighted parameter is the nature of the CL^+^. Through a comparison between the cell viability obtained with the liposome DOTAP/DOPE 1/1 and DC-Chol 1/1, it seems that the DC-Chol is more toxic than the DOTAP. Indeed, we show that the DOTAP/DOPE 1/1 can be used at higher N/P ratio or siRNA concentration without inducing any reduction in cell viability. Masotti et al. have not shown a difference in toxicity between the three cationic lipids that they tested (DOTAP, DC-Chol and DDAB), but they did not test an N/P ratio beyond 5, nor different siRNA concentrations. Finally, we can suggest that co-lipids, and especially the cholesterol, seem to influence cytotoxicity. Formulations containing cholesterol (DOTAP/Chol/DOPE 1/0.75/0.5 and 1/0.5/0.5) were more toxic. The cholesterol is usually added into liposomal formulations to increase the stability of the lipid bilayer [21]. Since, it seems that the cholesterol could be toxic and all formulations developed in this study were stable in times (data not shown), we can question its usefulness. Overall, we have demonstrated that the quantity of cationic liposomes and the quantity of siRNA induce a pronounced effect on cell viability. The nature and the quantity of CL^+^, as well as the presence of co-lipids, influence the toxicity of lipoplexes in a less flagrant manner.

Next, we evaluated the impact of lipid composition and N/P ratio on transfection efficiency. We have shown by flow cytometry that the proportion of lipoplexes stuck outside of the cellular membrane was proportional to the N/P ratio. Previously, we have demonstrated that a higher proportion of PEGylated lipoplexes blocked outside of the cell was attributed to a higher ζ-potential, and thus was attributed to a higher electrostatic interaction. Since the surface charge of selected formulations was similar in this study, we hypothesize that this phenomenon is due to a liposomes saturation of A549 cells. After the quenching of the external fluorescence, the percentage of transfected cells decreased by at least 30%. Furthermore, Wytrwal et al. explained that the fluorescence of fluorescein (like siCR-FITC) is quenched in acidic vesicles like endosomes or lysosomes [22]. This experiment allows us to quantify only the siRNA into the cytoplasm, free or still complexed to liposomes. Other studies showed that cellular uptake increased proportionally to the N/P ratio. However, since these studies do not distinguish internal and external fluorescence, the part of the signal corresponding to siRNA really present into the cytoplasm of cells cannot be known [20,23]. The interpretation of cellular uptake by flow cytometry using a fluorescent siRNA or lipid has to be done with caution.

The endosomal escape of siRNA and the complex dissociation were then evaluated using an active siRNA directed against a splicing factor expressed in A549 cells. First, we observe that the percentage of positive transfected cells revealed by flow cytometry was not correlated to the mRNA knockdown efficiency. Indeed, whereas liposomes DOTAP/Chol/DOPE 1/0.75/0.5 and 1/0.5/0.5 tended to induce the higher MFI, the highest mRNA extinction was obtained with formulation DOTAP/DOPE 1/1 and DC-Chol/DOPE 1/1. These results are in accordance with those obtained by Viricel et al. who developed new cationic switchable lipids. Depending on the lipids, they showed similar flow cytometry uptake profiles, whereas the knockdown of GFP in HeLa cells was very different [24,25]. They have proved by the addition of chloroquine, an endosomolytic agent, that endosomal entrapment is the major barrier to siRNA cytoplasmic delivery. In our study, we hypothesize that the dissociation of the complex intracellularly is the main hurdle to siRNA activity. Indeed, since we used fluorescein as a fluorescent probe, the MFI correspond to siCR-FITC only into the cytosol. If this intensity is not correlated to effectiveness of mRNA knock down, it means that siRNA is not free in the cytoplasm, but always complexed to its cationic carrier.

The highest siRNA effectiveness was obtained with liposomes DOTAP/DOPE 1/1 and DC-Chol/DOPE 1/1 complexed to siRNA at N/P ratio of 5 and 10. In these formulations, the cholesterol was removed whereas the proportion of DOPE increased. Ultimately, our results of mRNA extinction were logical, since the strategy used to provide the releasing of siRNA into the cytoplasm is the utilization of the pH-sensitive lipid DOPE [26]. Therefore, the higher proportion of DOPE provided a higher releasing of siRNA, and thus a higher protein knockdown. Finally, as stated previously, the dissociation between cationic liposomes and anionic siRNA seemed optimal, even at a higher N/P ratio as 10. Zhu et al. who synthetized polyplexes with a copolymer and siRNA showed that at an N/P ratio of 10 or 15, the electrostatic interaction was too strong inside the complexes, leading to lower siRNA efficacy [23]. We did not observe this phenomenon in our study, since the best mRNA knock down was obtained at an N/P ratio of 2.5, 5, and an N/P ratio of 10. 

In conclusion, our data elucidated the importance of different factors impacting on cytotoxicity and siRNA intracellular effectiveness when liposomes are used as carrier. The N/P molar ratio, as well as nucleic acid concentration, are crucial parameters which have to be optimized to develop safe lipoplexes. Moreover, the proportion of pH-sensitive lipid in lipidic formulation seems to be determinant for the release of siRNA into the cytoplasm of cells, and consequently, for the gene knockdown. Finally, the major observation is that a compromise between toxicity and efficacy should be found, regardless of the lipid composition of liposomes. Safe and efficient lipoplexes made of DOTAP or DC-Chol associated with DOPE were developed here and are proposed as a basis for the complexification of the system such as the addition of PEG or the introduction of ionisable CL^+^.

## 4. Materials and Methods

### 4.1. Materials

1,2-dioleoyl-3-trimethylammonium-propane (DOTAP), 1,2-dioleoyl-sn-glycero-3-phosphoethanolamine (DOPE) and 3ß-[*N*-(*N*′,*N*′-dimethylaminoethane)-carbamoyl]cholesterol hydrochloride (DC-Chol) and cholesterol (Chol) were purchased from Avanti Polar Lipids, Inc. (Alabaster, AL, USA). A549 cell lines were obtained from the American Type Culture Collection (ATCC, University Blvd, Manassas, VA, USA). Dulbecco’s Modified Eagle Medium (DMEM), Fetal Bovine Serum (FBS), MEM non-essential amino acids, Penicillin Streptomycin (PenStrep^®^), AmphotericinB (Fungizone^®^), Trypsine-EDTA and Opti-MEM^®^ were purchased from Gibco (Invitrogen, Rockville, MD, USA). Trypan blue (0.4% solution) was received from Sigma-Aldrich (Saint-Louis, MO, USA). siRNA control GL3 (siCR) and siRNA control-FITC (siCR-FITC), siRNA active against a splicing factor (siACT) [27,28] were provided by Eurogentec^®^ (Eurogentec SA, Liège, Belgium) with the following sense strand sequences: siCR 5′-CUUACGCUGAGUACUUCGAdTdT-3′; siCR fluo: 5′-AGAGUUCAAAAGCCCUUCAdTdT Fluorescein-3′. Resazurin sodium salt powder was obtained from Sigma-Aldrich (Schnelldorf, Germany). 

RNA isolation kit (NucleoSpin^®^ RNA) was obtained from Macherey-Nagel™ (GmbH & Co. KG, Berlin, Germany) and Transcriptor Reverse Transcriptase from Invitrogen™. SYBR green was provided by Nippon Genetics (Dueren, Germany). For the normalization, we used β-actin: forward primer, 5′-AGAAAACTGGCACCACACC-3′; reverse primer, 5′-AGAGGCGTACAGGGATAGCA-3′. Eurogentec^®^ provided all primers.

### 4.2. Preparation of Cationic Liposomes and Lipoplexes Formulations

Cationic liposomes were prepared by the hydration of lipid film method, as described previously [12,13]. As shown in Table 1, four different liposomes made of different lipids were formulated. A dry lipid film was prepared with correspondent lipids at a final lipid concentration of 5.6 mM and evaporated with a rotatory evaporator during 1 h at 30 °C (Buchi R200, BÜCHI Labortechnik GmbH, Hendrik-Ido-Ambacht, The Netherlands). The lipid film was rehydrated with RNAse free water, vortexed and directly extruded through polycarbonate membrane (400 nm and 200 nm). The cationic liposomes were then complexed to anionic siRNA by spontaneous electrostatic interaction during 30 min at room temperature. Different Nitrogen/Phosphate (N/P) ratios were tested: 0.25; 1.25; 2.5; 5; 7.5; 10. The concentration of siRNA is modified in function of the assay performed as explained in following sections. 

### 4.3. Physico-Chemical Properties: Size, Surface Charge and Complexation Efficiency

The Z-average size (nm) and the surface charge (mV) were measured at 25 °C with Malvern Zetasizer^®^ (Nano ZS, Malvern Instruments, Malvern, UK). Lipoplexes were prepared with 100 nM of siRNA control (siCR) in RNAse free water at a final volume of 1 mL. Moreover, the complexation between all liposomal formulations and siCR was evaluated with gel retardation assay, as described previously [12]. The N/P ratio at which complexation occurs was determined by agarose gel (4%) electrophoresis, and visualized with ethidium bromide (BET). Lipoplexes were prepared at different N/P ratios in RNAse free water (100 mL) to obtain a final siRNA concentration of 300 nM. These solutions were then loaded onto the agarose gel in TAE buffer, and the electrophoresis was performed at 100 V for 1 h in a Horizon 11.14 horizontal gel electrophoresis apparatus (Biometra, Goettingen, Germany). Gel was visualized by exposure to UV illumination by a Molecular Imager Gel Doc XR System (Bio-Rad, Hercules, CA, USA).

### 4.4. Cell Cultures

The human lung adenocarcinoma cell line A549 was used for in vitro tests. A549 cells were maintained in a DMEM medium supplemented with 10% FBS, 1% of MEM non-essential amino acids, 1% of PenStrep^®^ and 1% of Fungizone^®^, at 37 °C, in 5% CO_2_-humidified atmosphere. 

### 4.5. Cell Viability Assay

The cell viability of A549 was determined using Resazurin reagent after the treatment with lipoplexes formulations. A549 cells were seeded in 12-well plate at a density of 8 × 10^4^ cells/well. Lipoplexes were prepared in Opti-MEM^®^ at a final volume of 0.5 mL with the siCR at 40 nM and 100 nM. Cells were incubated during 4 h and the transfection was inhibited with 0.5 mL of medium containing 20% of FBS. Cells without any treatment were also seeded and considered as blank cells. 24 h post transfection, 90 µL of resazurin solution (0.15 mg/mL) were added into each well and incubated at 37 °C during 2 h. Then, 100 µL of each condition were added in triplicate into a 96-wells plate and measured with fluorescent spectrophotometer (Molecular Devices, SpectraMax.i3, Molecular Devices, LLC., CA, USA). The wavelengths of excitation and of emission were 560 nm and 590 nm respectively. DMEM media treated with resazurin in the same conditions were considered as background noise and untreated cells were considered as the control.

### 4.6. Cellular Uptake: Flow Cytometry Assay

Cellular uptake was quantified by flow cytometry on A549 cells. The cell line was seeded in 6-well plate (1.8 × 10^5^ cells/well), one day prior to transfection. The four formulations of liposomes were complexed to 40 nM of siRNA control-FITC (siFITC) (final volume of Opti-MEM^®^ of 1 mL). Following complexation, solutions were added dropwise on cells. Free siFITC was used as negative control. After 4 h of incubation, cells were washed, harvested with Trypsine-EDTA^®^ and re-suspended in 210 µL of PBS. Then, 90 µL of Trypan Blue^®^ was added to the solution during 5 min (final concentration of Trypan Blue^®^ is 1.2 mg/mL). Cells were washed with PBS and suspended in 300 µL before recording. 1.10^4^ cells were analysed by FACSCanto II flow cytometer and the data were processed by FACSDiva software (Becton and Dickenson, Mountain View, CA, USA).

### 4.7. Assessment of siRNA Efficiency by qRT-PCR

The endosomal escape of lipoplexes and the release of siACT into the cytoplasm were evaluated by following the knock-down of mRNA. Briefly, 1.8 × 10^5^ cells/well were seeded 24 h prior transfection. Lipoplexes were prepared with siACT and siCR at 40 nM in 1 mL of Opti-MEM^®^. After inhibition procedure with DMEM + 20% FBS and 48 h of incubation, cells were harvested and prepared in order to purify the cellular RNA with an RNA isolation kit. The RNA concentration was quantified using a NanoDrop1 ND-1000 Spectrophotometer (ISOGEN Life Science, De Meern, The Netherlands). Then, 1 µg of RNA and Transcriptor Reverse Transcriptase (InvitrogenTM) were employed to perform reverse transcription. Twnty five nanograms of cDNA were used for qPCR analysis (SYBR green) using the splicing factor primers and actin primers for the normalization. PCR was used with the following program: 50 °C 2 min; 95 °C 10 min; then 40 cycles of 95 °C 15 s; 60 °C 60 s; dissociation stage: 95 °C 15 s; 60 °C 30 s and 95 °C 15 s. To determine splicing factor mRNA expression, a comparative cycle (Ct) of one formulation with siACT was compared to the same formulation with siCR. Using the 2(-Delta Delta C(T)) method, splicing factor Ct values were normalized to actin Ct value using the formula: DCt = Ct sample − Ct Actin. Then, to determine the relative expression levels compared to siCR, we used: DDCt = DCt siCR − DCt sample, and to obtain the percentage mRNA remaining of sample: 2^DDCt [29].

### 4.8. Western Blot

A549 cells were treated in the same conditions as those described above. Seventy two hours post-transfected, cells were lysed with an SDS buffer (1% SDS; 40 mM Tris-HCl, pH 7.5; 1 mM EDTA and protease/phosphatase inhibitors). Equal aliquots of total cell protein extracts (30 µg) were separated by electrophoresis in 10% acrylamide gel, and transferred to a polyvinylidene difluoride membrane. Membranes were blocked for 1 hour in blotting-grade blocker (Bio-Rad) and hybridized to a protein-specific primary antibody. Membranes were washed three times for 10 min in 0.1% Tween 20 in 1 × PBS at room temperature, incubated with a secondary antibody HRP-conjugated, HRP-goat-anti-rabbit immunoglobulins (1:3000; Invitrogen, Carlsbad, CA, USA), for 1 h at room temperature. Detection was performed with Pierce ECL Western blotting substrate (Thermo Fisher Scientific, Waltham, MA, USA). BlueEasy prestained protein marker (Nippon Genetics) were used as size standards.

## Figures and Tables

**Table 1:** Physico-chemical characteristics of liposomes. Each value represents the mean (SEM) of four experiments (*n* = 4).

	Formulation	Molar Ratio	Z-Average Size (nm)	PDI	Surface Charge (mV)
1	DOTAP/Chol/DOPE	1/0.75/0.5	177.2 ± 5.1	0.10 ± 0.03	+56.2 ± 5.2
2	DOTAP/Chol/DOPE	1/0.5/0.5	181.4 ± 11.4	0.13 ± 0.07	+53.9 ± 4.7
3	DOTAP/DOPE	1/1	167.2 ± 4.2	0.07 ± 0.04	+56.7 ± 4.3
4	DC-Chol/DOPE	1/1	183.0 ± 4.6	0.06 ± 0.01	+53.3 ± 3.3

**Table 2:** Four formulations of cationic liposomes developed.

Formulation	Lipidic Composition	Molar Ratio	Cationic Lipid	% Age of Cationic Lipid	% Age of Chol	% Age of DOPE
1	DOTAP/Chol/DOPE	1/0.75/0.5	DOTAP	44	33	23
2	DOTAP/Chol/DOPE	1/0.5/0.5	DOTAP	50	25	25
3	DOTAP/DOPE	1/1	DOTAP	50	0	50
4	DC-Chol/DOPE	1/1	DC-Chol	50	0	50

## Supplementary Materials

The following are available online at http://www.mdpi.com/2079-4991/8/5/270/s1.

## Abbreviations

siRNA	small interfering RNA
RNAi	RNA interference
mRNA	Messenger RNA
DOTAP	1,2-Dioleoyl-3-trimethylammonium-propane (chloride salt)
DOPE	1,2-dioleoyl-sn-glycero-3-phosphoethanolamine
Chol	Cholesterol
DC-Chol	3ß-[*N*-(*N*′,*N*′-dimethylaminoethane)-carbamoyl]cholesterol hydrochloride
DMEM	Dulbeccos Modified Eagle Medium
N/P ratio	Nitrogen/Phosphate ratio
siCR	Control siRNA anti-GL3
siACT	siRNA anti-splicing factor
SF	Splicing factor
siFITC	Control siRNA labelled with FITC
FBS	Fœtal Bovine Serum
PBS	Phosphate Buffer Saline
MFI	Mean Fluorescence Intensity
BET	Ethidium bromide
Formulation 1	DOTAP/Chol/DOPE 1/0.75/0.5
Formulation 2	DOTAP/Chol/DOPE 1/0.5/0.5
Formulation 3	DOTAP/ DOPE 1/1
Formulation 4	DC-Chol/DOPE 1/1
PEG	Polyethylene glycol
PDI	Polydispersity Index
SEM	Standard error of the mean
ζ-potential	Zeta potential or surface charge
Ct	Comparative cycle
SNALPs	stable nucleic-acid-lipid particles
MCNPs	membrane/core nanoparticles
