# Supplementary material for: Cationic Liposomes Carrying siRNA: Impact of Lipid Composition on Physicochemical Properties, Cytotoxicity and Endosomal Escape
Source: Nanomaterials (Basel). 2018 Apr 24;8(5):270. doi: 10.3390/nano8050270 (PMC5977284; doi:10.3390/nano8050270)
Supplement: Supplementary file 1 [file nanomaterials-08-00270-s001.pdf]

Supplementary data

Figure S1. Polydispersity Index (PDI) of lipoplexes at different N/P molar ratios. Formulations DOTAP/Chol/DOPE 1/0.75/0.5, DOTAP/Chol/DOPE 1/0.5/0.5, DOTAP/ DOPE 1/1 and DC-Chol/DOPE 1/1 were complexed to siCR at 100nM at N/P ratios of 0.5, 1.25, 2.5, 5, 7.5 and 10 (n=4).

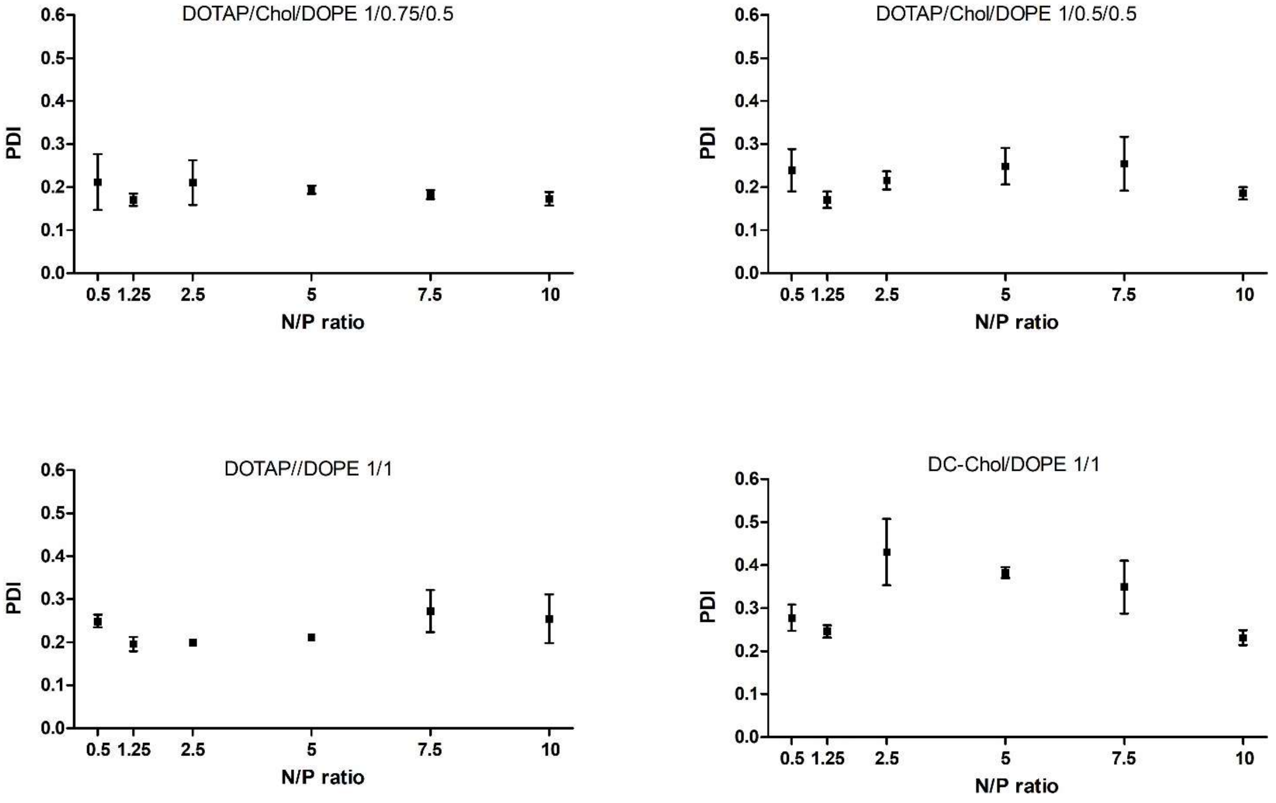

Figure S2. Cellular trafficking of lipoplexes on A549 cells, 4 hours post transfection. Mean fluorescence intensity (MFI) obtained after treatment with different formulations containing the siCR-FITC (40nM) by flow cytometry. Each condition is post-treated with Trypan Blue®. The dark line and percentage express the quantity of siRNA blocked outside of the cells (n =4).

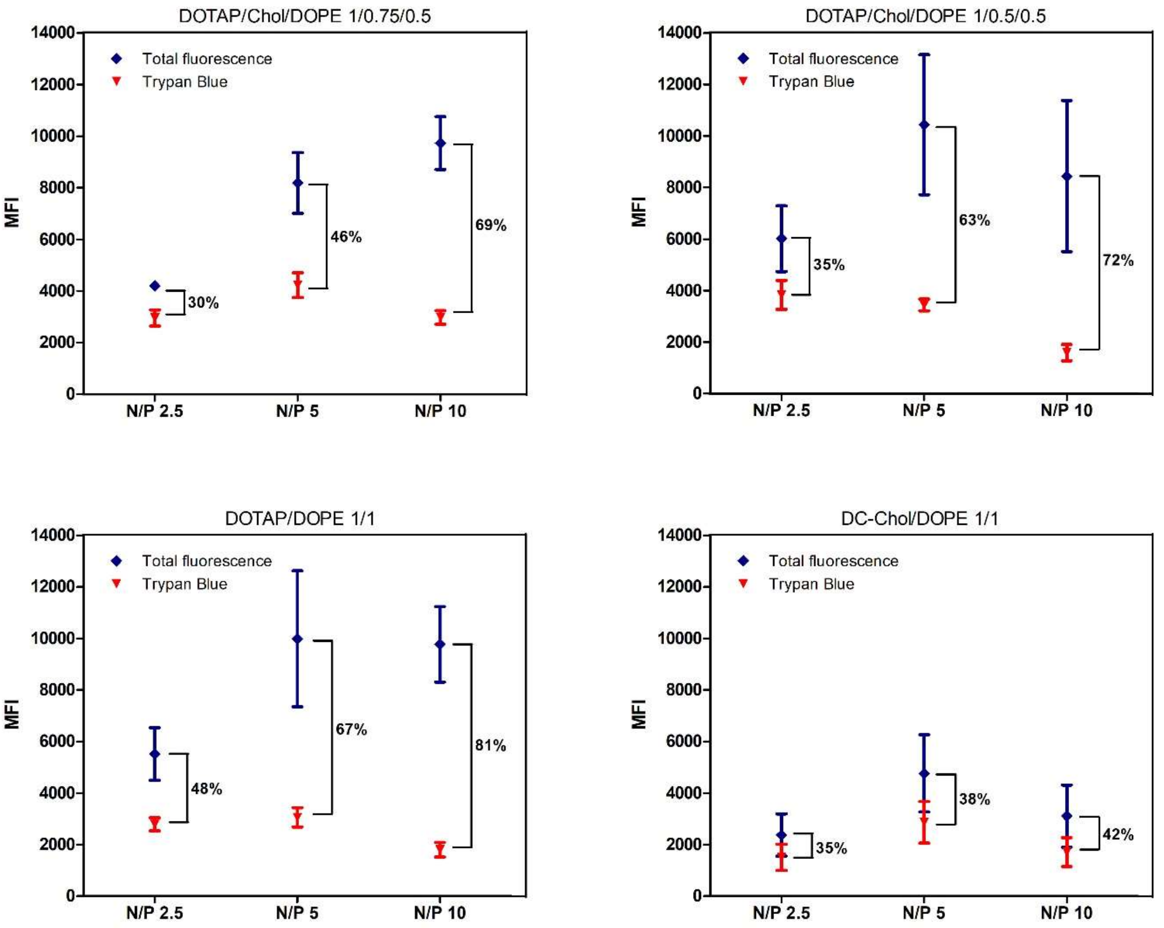
DOTAP/Chol/DOPE 1/0.75/0.5
DOTAP/Chol/DOPE 1/0.5/0.5
DOTAP/DOPE 1/1
DC-Chol/DOPE 1/1
Total fluorescence
Trypan Blue
MFI
N/P 2.5
N/P 5
N/P 10
30%
46%
69%
35%
63%
72%
48%
67%
81%
35%
38%
42%
